# Description of a new species of *Julolaelaps* (Acari, Mesostigmata, Laelapidae) from Iran

**DOI:** 10.3897/zookeys.526.5946

**Published:** 2015-10-12

**Authors:** Alireza Nemati, Elham Riahi, Dariusz J. Gwiazdowicz

**Affiliations:** 1 Department of Plant Protection, Faculty of Agriculture, University of Shahrekord, Iran; 2 Department of Entomology, Agricultural College, Tarbiat Modares University, Tehran, Iran; 3 Poznan University of Life Sciences, Faculty of Forestry, Wojska Polskiego 71C, 60–625 Poznań, Poland

**Keywords:** Laelapidae, Taxonomy, *Julolaelaps*, Iran

## Abstract

*Julolaelaps hallidayi* **sp. n.**, was collected from soil of river verge in Brujen, Chaharmahal va Bakhtiari province, Iran. Description and illustrations of this new species based on adult females are presented. Some entries are added to the key of Moraza and Kazemi (2012) to include the new species.

## Introduction

Evolutionary relationships between mites and other arthropods date back to approximately 100 million years ago (Southwood 1973). As more niches became available, mites developed a wide variety of well-known symbiotic relationships with many arthropods (Lindquist 1975) including many species in the insect orders Coleoptera, Diptera, Hymenoptera, and Lepidoptera, and also with other arthropods such as myriapods (Farfan and Klompen 2012). The laelapid subfamily Iphiopsidinae *sensu* Evans (1955) was promoted to family level (Iphiopsididae) by Casanueva (1993) based on phylogenetic studies. The main character differentiating this family and Laelapidae is the lack of seta *pl2* on genu IV in iphiopsidids (Casanueva 1993). The Iphiopsididae includes three subfamilies and ten genera of mites that are associated with millipedes, centipedes, spiders, and terrestrial crustaceans. There is little information on the biology of iphiopsidids, although based on the regressive nature of the characters it seems that they have a paraphagic or parasitic mode of life on their terrestrial arthropod hosts (Lindquist et al. 2009).

*Julolaelaps* was erected by Berlese (1916) for a small group of mites living on Julids. In the definition of the genus he states that the species resemble very closely those of the genus *Hypoaspis* but lack claws on all legs (Evans 1955). Vitzthum (1941) referred to *Hypoaspis* Canestrini, and *Julolaelaps* Berlese as members of the subfamily Hypoaspidinae and *Iphiopsis* Berlese and *Jacobsonia* Berlese as members of the Iphiopsinae. Evans (1955) noted the possible absence of claws in all legs of *Julolaelaps* (present in most *Hypoaspis*) as a generic character. Ryke (1959) introduced *Julolaelaps* as a subgenus of *Hypoaspis*, and described three new species while referring to the presence of small claws on leg I ambulacra. Maes (1983) described four additional species of *Julolaelaps*, as a separate genus, and confirmed the presence of reduced claws on leg I.

Most *Julolaelaps* species that have been reported until now are associated with small millipedes (Berlese 1916, Maes 1983, Fain 1987, Uppstrom and Klompen 2005, Kontschan 2005, Salmane and Telnov 2007, Moraza and Kazemi 2012), and a few associated with Polydesmida (Ishikawa 1986). The feeding habitats (parasitism or paraphagy) of *Julolaelaps* are not confirmed (Salmane and Telnov 2007). Moraza and Kazemi (2012) presented a key for this genus based on known females and males, agreed the idea of Ryke (1959) to consider Laelaps (Hypoaspis) indicus Vitzthum as a synonym of *Julolaelaps luctator* Berlese, 1916. The present paper is devoted to the description of a new species of *Julolaelaps*, found in the soil of a river verge in Brujen, Chaharmahal va Bakhtiari province, Iran, followed by a short discussion regarding the status of correct family for this genus.

## Materials and methods

Mites were collected in soil from Brujen, Chaharmahal va Bakhtiari province in Iran, extracted from samples using Berlese-Tullgren funnels, placed in lactic acid at 55 °C for clearing and then mounted in Hoyer’s medium on permanent microslides for microscopic examination. Line drawings were made by use of a drawing tube and figures were performed with Corel X-draw software, based on the scanned line drawings. Measurements of structures are expressed as minimum-maximum ranges in micrometers (µm). The dorsal setae notation followed that of Lindquist and Evans (1965). Leg and pedipalp setal notation and chaetotactic formulae are based on Evans (1963a, b respectively). Terminology for idiosomal glands and lyrifissures follows Johnston and Moraza (1991). We have attempted to identify all pore-like structures, but acknowledge that some might have been overlooked. Length of the dorsal shield is the distance from its antero-median edge anterior to bases of setae *j1* to its postero-median edge posterior to bases of setae *Z5*; width of dorsal shield was measured at widest part; length of the sternal shield was measured along midline from anterior edge to its posterior margin, width measured between coxae II-III (widest point) and slightly above the insertion of *st2* (narrowest point); the length of anal shield is midline from the anterior margin to the posterior edge of the cribrum, and width was measured at widest point. Setae were measured at level of insertions to their tips and distance between setae as the distance between their insertions. Length of leg segments was measured dorso-medially, and tarsi were measured excluding the stalk and its appendages.

## Description

### Julolaelaps hallidayi sp. n.

Taxon classificationAnimaliaMesostigmataLaelapidae

http://zoobank.org/98513061-C2DA-41B3-80DF-E0A373F2053A

Figures 1–2 , 3–5 , 6–9 , 10–13

#### Specimens examined.

Holotype, female, Brujen region, Chaharmahal va Bakhtiari province, Iran, soil, coll., B. Jalili, 2011; paratype, female (same data as holotype): deposited in Acarological Laboratory of Shahrekord University, Chaharmahal va Bakhtiari province, Iran; paratype, female collected from soil, Shahreza, Esfahan province, coll., F. Shameli, 2014: deposited in the Senckenberg Museum fur Naturkunde Görlitz, Germany.

#### Note.

Some unknown arthropods species such as members of Thysanura, Microcoryphia, Diplopoda and Chilopoda were separated associated with the soil which contained specimens of *Julolaelaps* species.

#### Diagnosis.

Medium sized laelapid mite; with 33 pairs of simple acicular setae on dorsal shield, setae *z1, z3, z6, r4*, and *r6* missing in podonotal part, without extra setae between *J* and *Z* series; pre-sternal area not sclerotized; genital shield with reticulated pattern possess seven closed cells with eight small indentations at their margins, cells surrounded antero-laterally by inverse V shaped lines; peritremes short, extending to posterior margin of coxae II; tibia I and III with two *pl* and one *al* respectively.

**Figures 1–2.**
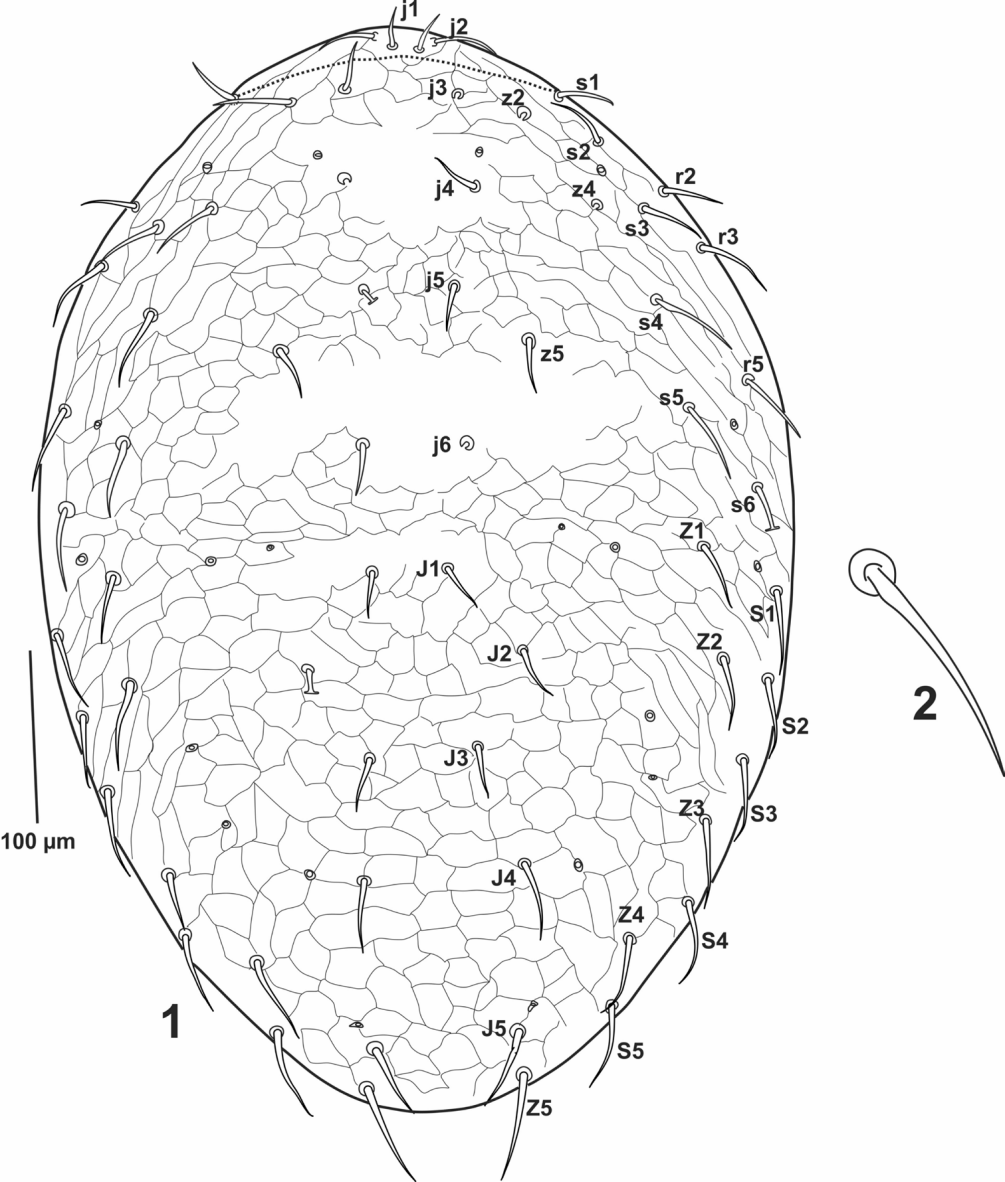
*Julolaelaps hallidayi* sp. n. (female): **1** Dorsal idiosoma **2** Example of dorsal setae.

***Dorsal idiosoma***. Dorsal idiosoma oval-shaped (Fig. 1), dorsal shield covered all dorsal surface, polygonal reticulation distinct on whole dorsum except of small area around *j4* and area between *z5* and *j6*. Dorsal shield 489-567 long, 341-348 wide between of setae *r3-5* (n = 3), with 33 pairs of simple acicular setae (Fig. 2), 18 pairs (*j1-6, z2, z4-5, s1-6* and *r2-3, r5*) located on podonotum, *z1, z3, z6, r4*, and *r6* missing on dorsal shield in podonotal part, and 15 pairs on opisthonotum (*J1-5*, *Z1-5* and *S1-5*) without extra setae between *J* and *Z* series. Dorsal setae length: *j1* (16-18), *j2-6* (20-31), *z2* and *z4* (34-39), *z5* (26), *s1-6* (29-39), *r2-3, r5* (29-36), *J1-5* (20-34), *Z1-5* (29- 42), *S1-5* (34-39). Cuticle between dorsal and ventral side of body bent down on ventral side, and bearing *R1* (18-21), *R2* (21-23), *R4* (23-29), *R5* (23-26), *R6* (26-29), and *UR1-2* (23-26). Podonotal part with three and opisthonotal region with seven pairs of discernible pore-like structures, as shown in figure 1; however, it is acknowledged that some might have been overlooked.

**Figures 3–5.**
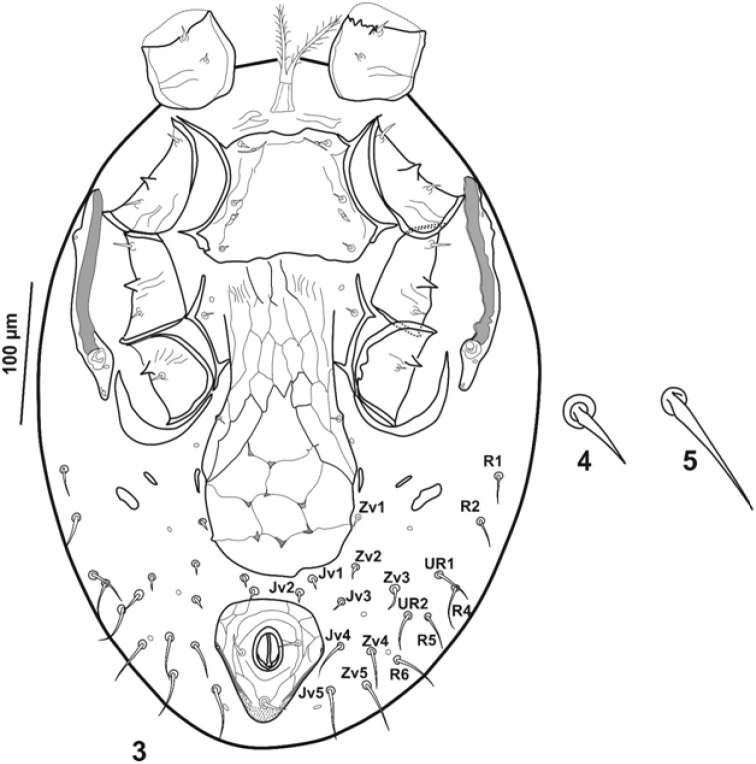
*Julolaelaps hallidayi* sp. n. (female): **3** Ventral idiosoma **4–5** Examples of ventral setae.

***Ventral idiosoma*** (Fig. 3). Tritosternum with columnar base, 18–21 long, and pilose laciniae (62). Pre-sternal area not sclerotized, with lines striation. Sternal shield with thin line reticulation in lateral surfaces, 96–99 long, 148–151 wide (at level of projection between coxae II-III) and 99 slightly above the insertion of *st2* (narrowest point), antero-medially slightly concave, posterior margin irregular. Sternal setae very short, *st1-3* (4), smooth, with conspicuous alveoli, *iv1* slit-like, located slightly behind *st1*, *iv2* slit-like, between *st2-st3*. Metasternal plates absent. Setae *st4* (5) and pore-like *iv3* located on integument posterior to sternal shield and interior to endopodal plates III/IV. Tongue-shaped genital shield 205–211 µm long (excluding hyaline flap at base of posterior margin of sternal shield), 83-88 wide at level of *st5*, and 101 at widest part near para-genital platelets, the ratio of length to width (L/W) is 2.32 /2.53 (width at level of *st5*), bearing one pair of setae (*st5* = 4-5) and reticulated pattern with seven closed cells with eight small indentations at their margins, cells surrounded antero-laterally by inverse V shaped lines (Fig. 3), genital shield separated from anal shield by about the length of the anal opening, paragenital pore-like opening on soft integument between genital seta and coxa IV. Subtriangular anal shield reticulated, anterior margin semi-circular, 78–83 long, and 73–75 wide, with one pair of minute adanal gland pores (*gv3*) on lateral margins, paranal setae (10) slightly smaller than post-anal seta (13). Cribrum extending laterally slightly upper than the level of post-anal seta insertion. Opisthogastric surface with one pair of suboval metapodal plates, one pairs of minute platelets (between metapodal plate and para-genital platelet), one pair of narrow, slightly elongate para-genital platelets, smooth setae *Jv1-3* (6-8), *Jv4* (18-23), *Jv5* (26), *Zv1* (5-7), *Zv2* (8-10), *Zv3* (13-16), *Zv4* (23-26), *Zv5* (26-29), (Figs 4–5), and five pairs of pore-like structures. Stigmata located in anterior level of coxa IV surrounded by nearly narrow stigmatal plate. Peritremes short, extending to posterior margin of coxae II, peritrematal plate wider in anterior part, and with one glandular poroid *gp* (Fig. 3), separated from exopodal shield. Small poststigmatal plate with two pores. Exopodal plates like a narrow crescent-shape strip expanded posteriad coxae IV. Endopodal plates II/III fused to lateral margins of sternal shield, and III-IV elongate, narrow and angular.

**Figures 6–9.**
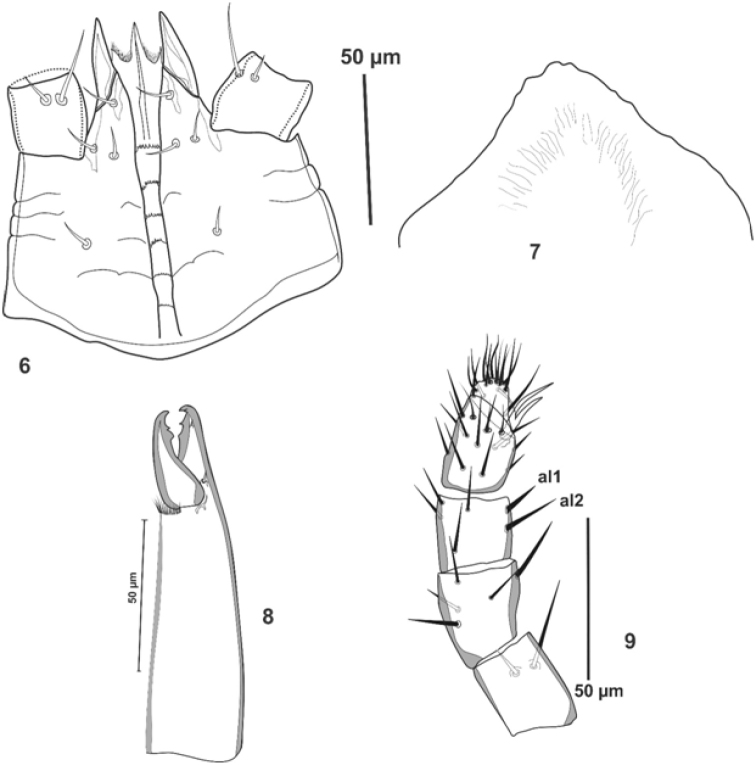
*Julolaelaps hallidayi* sp. n., (female): **6** Subcapitulum **7** Epistome **8** Chelicera **9** Palp.

***Gnathosoma***. Hypostome (Fig. 6) with three pairs of smooth simple setae; *h1-3* (8-10), palpcoxal setae 8-9 long. Deutosternal groove with six rows of multi-dentate (6-8 teeth), the denticles tend to be smaller from anterior to posterior rows. Corniculi normal (30-32), horn-like. Epistome with nearly smooth rounded anterior margin (Fig. 7). Cheliceral arthrodial processes crownet-like (Fig. 8), movable digit (26-29) with two teeth in addition to apical tooth, middle article 75-78 long, ending in fixed digit (29-31), bearing two teeth in addition to terminal tooth and very short setaceous pilus dentilis. Palp chaetotaxy normal for the free-living forms (sensu Evans and Till 1965), with simple and thin setae except *al* on femur, and *al1* and *al2* on genu slightly thickened; palp-tarsal claw two-tined, basal tine smaller (Fig. 9).

**Figures 10–13.**
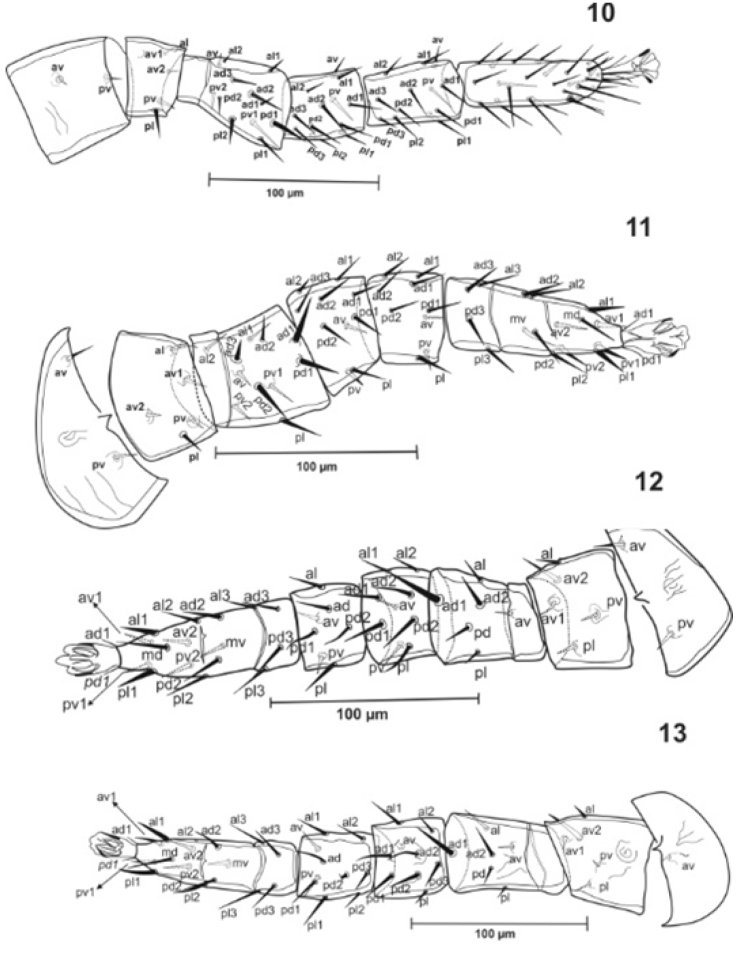
*Julolaelaps hallidayi* sp. n. (female): **10** Leg I **11** Leg II **12** Leg III **13** Leg IV.

***Legs***. Tarsi I-IV with small and not well sclerotized claws, the sclerotization status is more distinct in their tips (Figs 10–13). **Leg I** 374, coxa (70-73), trochanter (29-34), basi-femur (18-21), telo-femur (42), genu (42-47), tibia (55-60), tarsus (107-112); **leg II** 278-302 coxa (34-47), trochanter (31-39), basi-femur (16), telo-femur (34-39), genu (36-44), tibia (39), tarsus (75-91); **leg III** 307, coxa (36-39), trochanter (52), basi-femur (23), telo-femur (34), genu (31-36), tibia (34-36), tarsus (91); **leg IV** 359-385, coxa (39-47), trochanter (65-70), basi-femur (18-23), telo-femur (47-52), genu (39), tibia (44), tarsus (107-109). Legs I and IV longer than legs II and III. All leg setae smooth and pointed. Chaetotaxy of legs is as follows: **Leg I**: coxa 0 0/1 0/1 0; trochanter 1 0/2 1/1 1 (*pl* and *pv* slightly thickened); femur 2 3/12/2 2 (*ad2*, *pd1* and *pl2* slightly thickened); genu 2 3/1 3/1 2; tibia 2 3/1 3/1 2 (Fig. 10). **Leg II**: coxa 0 0/1 0/1 0; trochanter 1 0/2 0/1 1; femur 2 3/1 2/2 1 (*ad1*, *ad3*, *pd1-2* and *pl* slightly thickened); genu 2 3/1 2/1 1; tibia 2 2/1 2/1 1; tarsus 3,3/2,3/2,3 + *mv*, *md* (*al1*, *av1-2*, *pl1* and *pv1*-2 more thickened than the others) (Fig. 11). **Leg III**: coxa 0 0/1 0/1 0; trochanter 1 0/2 0/1 1; femur 1 2/1 1/0 1(*ad1* thickened and *ad2* slightly thickened); genu 2 2/1 2/1 1; tibia 1 1/1 2/1 1; tarsus 3 3/2 3/2 3 + *mv*, *md* (*al1*, *pv1* and *pl1* thickened). **Leg IV**: coxa 0 0/1 0/0 0; trochanter 1 0/2 0/1 1 (*av2* slightly thickened); femur 1 2/1 1/0 1 (*ad1* slightly thickened) (Fig. 13); genu 2 2/1 3/0 1; tibia 2 1/1 3/1 2; tarsus 33/23/23 + *mv*, *md* (*al1* and *pl1* slightly thickened).

***Insemination structures***. Not discernible.

***Male***. Unknown.

#### Etymology.

This species is named in honour of Dr. Bruce Halliday (CSIRO Ecosystem Sciences, Canberra, Australia).

#### Remarks.

The species of the genus *Julolaelaps* having been reported so far can be divided into two groups: the first group consisting of 14 species with reduced number of setae on dorsal shield than *Julolaelaps hallidayi* sp. n. (9–23 pairs), and the other with more than 29 pairs of dorsal setae (*sensu* Moraza and Kazemi 2012), which comprises seven species including *Julolaelaps luctator* Berlese, 1916, *Julolaelaps dispar* Berlese, 1916, *Julolaelaps pararotundatus* Ryke, 1959, *Julolaelaps spirostrepti* Oudemans, 1914, *Julolaelaps tritosternalis* Moraza and Kazemi 2012, *Julolaelaps moseri* Hunter & Rosario, 1986, and *Julolaelaps hallidayi* sp. n. Except for *Julolaelaps tritosternalis*, the number of dorsal setae in the above-mentioned species is higher than in *Julolaelaps hallidayi* sp. n. The two latter species are different from each other in that the former has 32 pairs of dorsal setae, while the latter has 33 pairs. Furthermore, the main discrepancy between them refers to the presence of *S1* in *Julolaelaps hallidayi* sp. n., and absence of these structures in *Julolaelaps tritosternalis*. In addition, *Julolaelaps tritosternalis* has a disc-like structure on the base of tritosternum, while that structure is not present in *Julolaelaps hallidayi* sp. n. Leg chaetotaxy of *Julolaelaps hallidayi* sp. n., is different from that of *Julolaelaps tritosternalis* Moraza & Kazemi, 2012: tibia I and III in *Julolaelaps hallidayi* sp. n. bears two *pl* and one *al* while in *Julolaelaps tritosternalis* tibia I and III are with one *pl* and two *al*.

## Discussion

The loss of seta *pl2* on genu IV in iphiopsidids phylogenetically defines the family as an entity separate from the Laelapidae (Casanueva 1993), but its laelapid roots may clearly be seen in the genus *Julolaelaps*, an assemblage of iphiopsidine millipede associates that had long been considered a subgenus of the broadly defined laelapid genus *Hypoaspis* (Lindquist et al. 2009, Ryke 1959).

Based on Casanueva (1993) study, Iphiopsididae was recognized as a separate family from Laelapidae by considering two phylogenetic attributes: lack of seta *av-2* on tibia I in the Iphiopsididae, and lack of seta *pl-2* on genu IV in the Laelapidae. Assigning the new species to the family Iphiopsididae does not fit properly based on the above-mentioned attributes. In the first instance, *Julolaelaps hallidayi* sp. n. is defined by one apomorphic character (lack of postero-lateral seta *pl2* on genu II), which has also evolved in group I (Pseudoparasitini) of the Laelapidae. Furthermore, *Julolaelaps hallidayi* sp. n. presents one synapomorphic character, which is a regressive autapomorphy, supporting groups I and II of the Laelapidae: lack of setae *pv1* on genu IV. In addition, two synapomorphic characters of *Julolaelaps hallidayi* sp. n., the loss of setae *pl2* on genu IV and the absence of podonotal setae *r6*, are shared with groups I-II and IV of Laelapidae, respectively. Finally *Julolaelaps hallidayi* sp. n., along with some other species of the genus *Julolaelaps*, emerges from the subfamily Iphiopsidinae Kramer (Casanueva 1993) by lacking two synapomorphic characters: a reduced hypostomal process and the presence of additional setae (*px*) between *J* and *Z* series, as well as two apomorphic characters (loss of hypostomal setae *h1* or *h3* on the gnathosoma and absent peritreme).

On the other hand, Lindquist et al. (2009) accepted the idea of Casanueva (1993) to consider iphiopsidids as members of a separate family from laelapid mites by referring to some characters: tibia I usually with one ventral seta, lacking seta *av2*; genu IV usually with one postero-lateral seta, lacking seta *p12*; subcapitulum with internal malae usually weakly developed, with nearly smooth lateral margins and shorter than corniculi, which is discussed below. However species of laelapid mites usually possess setae *av2* on tibia I (Beaulieu 2009, Faraji and Halliday 2009, Evans and Till 1965, 1966, Kavianpour et al. 2013, Lindquist et al. 2009, Nemati and Kavianpour 2013, Nemati and Mohseni 2013), but Moraza and Kazemi (2012) considered different groups in *Julolaelaps* species assemblage. Within species with edentate chelicerae in males, one group includes species with largely complete dorsal complement of setae and usually with strong neotrichy in dorsal setae on soft cuticle, a well-developed genital shield, wider than anal shield (except *Julolaelaps luctator*), usually long peritremes (extending at least to anterior margin of coxa II), and seta *av-2* present in tibia I. So, some species of *Julolaelaps* possess seta *av2* on tibia I and this character cannot be considered as an apomorphic feature for iphiopsidids. Furthermore, loss of seta *pl2* on genua IV is a character for laelapid mites and iphiopsidids mites also exhibit this character (Beaulieu 2009, Faraji and Halliday 2009, Kavianpour et al. 2013, Moraza et al. 2009, Nemati and Kavianpour 2013, Nemati and Mohseni 2013, see also above explanations). In addition, Moraza and Kazemi (2012) described *Julolaelaps tritosternalis* with subcapitular internal malae well developed, with lateral margins fimbriated and longer than corniculi.

In this research we are following Maes (1983) and Moraza and Kazemi (2012) in keeping the *Julolaelaps* as a separate genus of the family Laelapidae Berlese, 1882, subfamily Iphiopsidinae Kramer, 1886.

This research has posed questions which are in need of further investigation, and considerably more work is needed to determine the level of Iphiopsididae or Iphiopsidinae as well as the name of genera that will be categorized within that level.

### Modified key couplet to the species of *Julolaelaps* (after Moraza and Kazemi 2012), with emendations to add *Julolaelaps hallidayi* sp. n.

**Table:** 

6	Dorsal shield with 36 pairs of setae; setae *z1*, *z6* and *S1* present; setae *Z5* twice as long as *j1*; strong neotrichia on series *R*	***Julolaelaps moseri* Hunter & Rosario**
–	Dorsal shield with 32-33 pairs of setae; setae z1, z6, r4, r6 absent and *S1* present or absent	**7**
7	With 32 pairs of dorsal shield setae; *S1* absent; tritosternal base with ventral disc-like structure	***Julolaelaps tritosternalis* Moraza & Kazemi**
–	With 33 pairs of dorsal shield setae; *S1* present; tritosternal base normal and lacks ventral disc-like structure	***Julolaelaps hallidayi* sp. n.**

## Supplementary Material

XML Treatment for Julolaelaps hallidayi
